# Isolation and biochemical characterization of bradykinin-potentiating peptides from *Bitis gabonica rhinoceros*

**DOI:** 10.1186/s40409-017-0124-9

**Published:** 2017-06-26

**Authors:** Tamara M. Fucase, Juliana M. Sciani, Ingrid Cavalcante, Vincent L. Viala, Bruno B. Chagas, Daniel C. Pimenta, Patrick J. Spencer

**Affiliations:** 1 Biotechnology Center, Nuclear and Energy Research Institute (IPEN), Av. Lineu Prestes, 2242, São Paulo, SP CEP 05508-000 Brazil; 2 Laboratory of Biochemistry and Biophysics, Butantan Institute, Av. Vital Brasil, 1500, São Paulo, SP CEP 05503-900 Brazil

**Keywords:** Peptide, Hypotension, Viperinae

## Abstract

**Background:**

Venoms represent a still underexplored reservoir of bioactive components that might mitigate or cure diseases in conditions in which conventional therapy is ineffective. The bradykinin-potentiating peptides (BPPs) comprise a class of angiotensin-I converting enzyme (ACE) inhibitors. The BPPs usually consist of oligopeptides with 5 to 13 residues with a high number of proline residues and the tripeptide Ile-Pro-Pro (IPP-tripeptide) in the C-terminus region and have a conserved N-terminal pyroglutamate residue. As a whole, the action of the BPPs on prey and snakebite victims results in the decrease of the blood pressure. The aim of this work was to isolate and characterize novel BPPs from the venom of *Bitis gabonica rhinoceros*.

**Methods:**

The crude venom of *B. g. rhinoceros* was fractionated by size exclusion chromatography and the peptide fraction (<7 kDa) was separated by reverse phase chromatography (RP-HPLC) and analyzed by ESI-IT-TOF-MS/MS. One new BPP was identified, synthetized and assayed for ACE inhibition and, in vivo, for edema potentiation.

**Results:**

Typical BPP signatures were identified in three RP-HPLC fractions. CID fragmentation presented the usual y-ion of the terminal P-P fragment as a predominant signal at m/z 213.1. *De novo* peptide sequencing identified one *Bothrops*-like BPP and one new BPP sequence. The new BPP was synthesized and showed poor inhibition over ACE, but displayed significant bradykinin-induced edema potentiation.

**Conclusions:**

So far, few BPPs are described in Viperinae, and based on the sequenced peptides, two non-canonical sequences were detected. The possible clinical role of this new peptides remains unclear.

## Background

Snake venoms are a complex mixture of peptides and proteins, including peptidases. Many of these toxins mimic structurally and functionally endogenous molecules of the prey involved in homeostatic processes, escaping, however, from the regulation mechanisms, therefore disturbing physiological equilibrium. Besides snake venoms proteomics, several authors are now focusing on peptidomics with the aim of isolating new potential drugs [1–5]. Unlike proteins, peptides have the advantage of being small, easily synthetized and presenting low immunogenicity [6]. Due to their high degree of target specificity, venom toxins have been increasingly used as lead compounds in the development of drugs [7]. Biologically active proteins and peptides, as those found in venoms, may have a potential therapeutic use for the correction of hemostatic disorders and cellular adhesion among other applications [8]. Bradykinin (BK), which was first discovered by Rocha and Silva et al. in 1949 [9], can be described as the hydrolysis product of high molecular mass kininogen by plasma kallikrein [10, 11]. This molecule has been associated to several physiological processes such as the inflammatory responses and induction of nociception and hyperalgesia [12].

The bradykinin-potentiating peptides (BPPs) comprise a class of angiotensin-I converting enzyme (ACE) inhibitors [13]. The somatic ACE is a dipeptidyl carboxypeptidase situated on the external surface of endothelial cells. This enzyme plays a central role in blood pressure regulation, is composed of two highly similar domains, the N- and C-domains [14]. Each one of them contain an active site, characterized by the presence of a zinc-metallopeptidase HEXXH consensus motif [15, 16]. ACE increases blood pressure by generating angiotensin II (Ang II) from angiotensin I and by inactivating BK [17]. The C-domain of mammalian ACE is mainly responsible for Ang II formation while BK is inactivated by both domains with the same efficiency [18].

Many BPPs were described in the venoms from arthropods, amphibians and snakes, most of them being ACE inhibitors [19]. In the case of BPPs isolated from snake venoms, these molecules are recognizable by a common structural pattern (Pyr-EXnPXPXIPP) – where Pyr is pyroglutamic acid and X is any amino acid residue whreas Cys, with the C-terminus sequence PXIPP, is crucial for the binding in the ACE catalytic site [7, 13].

Thereafter, BPPs were isolated from many snake venoms and shown to potentiate contraction in the isolated guinea pig ileum and to increase the hypotensive effect of BK, by inhibiting BK degradation [20]. The hypotensive effects of these BPPs were also associated with the inhibition of the conversion of angiotensin I to its active metabolite Ang II [21]. These crucial findings paved the way for the later development of ACE inhibitors, such as Captopril®, for the treatment of hypertension and heart failure [22, 23]. In the present work, we describe the sequence of two BPPs (one new and one already described for *Bothrops jararaca*) isolated from the venom of *Bitis gabonica rhinoceros* and present an in vivo functional characterization of the synthetic analogue. The new BPP was termed BPP-10 g-AP.

## Methods

### Animals

Male Wistar (200 g-250 g) rats, bred at the Nuclear and Energy Research Institute (IPEN), São Paulo, SP, Brazil were used for the in vivo assays. The animals had access to food and water ad libitum, and were kept under a 12 h-light/dark cycle. Procedures involving animals and their care were in accordance with the guidelines for the use of animals on biomedical research and were approved by the Animal Ethics Committee (protocol 171/16) of IPEN.

### Drugs and reagents

Acetonitrile (HPLC grade) was purchased from J. T. Baker (USA). Laboratory-deionized water was produced by a Milli-Q water purifying system (Millipore, USA), iodoacetamide, somatic ACE (rabbit lung) and BK acetate were purchased from Sigma-Aldrich (USA). The synthetic peptide APQERGPPEIPP was purchased from FastBio Ltda (Brazil).

### Peptide purification

Crude venom of *B. g. rhinoceros* (30 mg) was fractionated by size exclusion chromatography (SEC) on a Superdex 75 column at pH 7.0, using 0.1 M ammonium bicarbonate buffer. The flow rate was of 0.6 mL/min. Based on retention times, the peaks with molecular mass lower than 7 kDa were individually pooled and lyophilized.

**Fig. 1:**
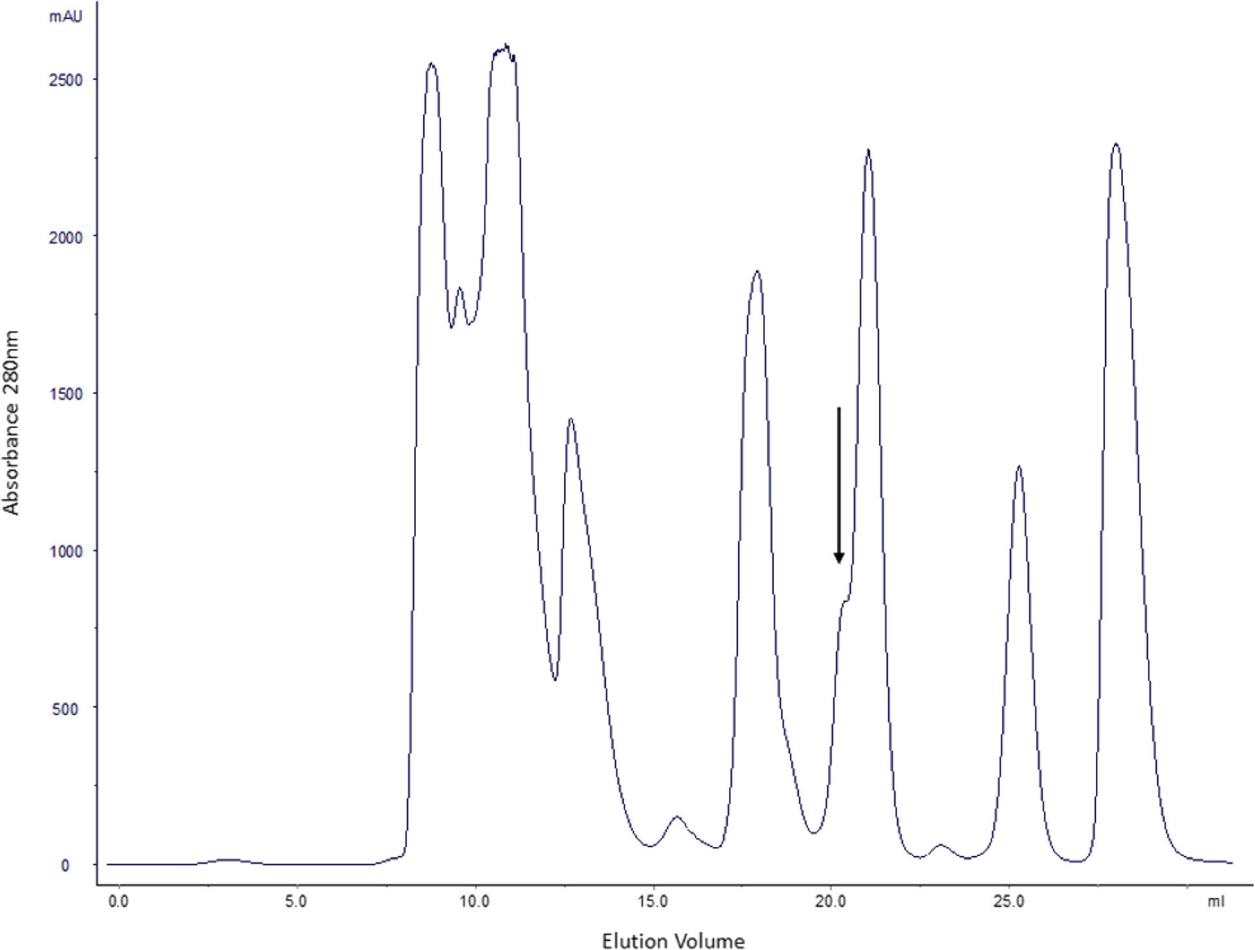
Elution profile of 30 mg of *B. g. rhinoceros* venom on a Superdex 75 10/300 column. The flow rate was of 0.6 mL/min. The *arrow* indicates the fraction (peak 9) that was further analyzed

The peak of interest (peak 9, Fig. 1) was injected in a C_18_ column (4.6 × 150 mm Sigma- Aldrich) using 0.1% (v/v) trifluoroacetic acid (TFA)/water (solution A) and 90% acetonitrile/0.1% TFA/water (solution B). Prior to injection, the column was equilibrated with 20% B. Elution was performed with a gradient of B solution (90% acetonitrile/0.1% TFA/water) ranging from 20 to 50%, in 20 min, at a flow rate of 1 mL/min. The peaks were manually collected.

### *De novo* peptide sequencing

For mass spectrometric *de novo* peptide sequencing the samples were directly injected on the ESI-IT-TOF (Shimadzu Co., Japan), at 0.05 mL/min constant flow rate, in positive mode, for MS, MS^2^ and MS^3^ analyses. The interface voltage was kept at 4.5 kV, the detector voltage at 1.8 kV and the capillary temperature at 200 °C. Data were collected at a range of 50-1800 m/z. For fragmentation, the precursor ions were selected under a 0.5 m/z window, and the argon collision energy was kept at 50%. The instrument control and data acquisition were performed with the LC-MS Solutions software (Shimadzu Co., Japan).

### ACE inhibition assay

ACE inhibition was performed by continuously monitoring the hydrolysis of the fluorescence resonance energy transfer (FRET) substrate Abz-FRK(Dnp)P-OH (ο-aminobenzoic acid-Phe-Arg- Lys(DNP)-Pro-OH), in the presence or absence of the synthetic inhibitor, as described by Carmona et al. [24]. Briefly, 0.5 mU of ACE (1 mU = nmol of substrate hydrolyzed per min) was added to a buffered (100 mM Tris–HCl, 50 mM NaCl and 10 mM ZnCl 2, pH 7.0) 2 μM substrate solution, and the fluorescence (λ_ex_ = 320 nm, λ_em_ = 420 nm) was recorded after 5 min in the absence of the inhibitor. This value was considered V_0_. Increasing concentrations of the inhibitor were then added every 5 min and the fluorescence values were recorded. The inhibition constant was then calculated according to Carmona et al. [24].

### Bradykinin-potentiating activity in vivo

Edema was induced by intraplantar injection of 2 ng of BK diluted in 50 μL saline into the left hind paw, which was measured every 5 min for 40 min by a plethysmograph (H. Basile, Italy). The right paw was injected with 50 μL mL saline (NaCl 0.9% w/v). In parallel, a group was injected with the synthetic potentiating peptide (40 ng/mL) in the left paw 10 min before the injection of BK, in order to evaluate the potentiation of BK. In both cases, the volume of the right paw was subtracted from that of volume of the left paw, to give net edema, expressed in μL.

### Statistical analysis

One-way analysis of variance (ANOVA) was performed. The significance level was considered as p < 0.05.

## Results

### Purification and characterization of the peptide

The gel filtration of the crude venom resulted in eleven peaks (Fig. 1). The low molecular mass peak indicated by the arrow was pooled and lyophilized.

**Fig. 2:**
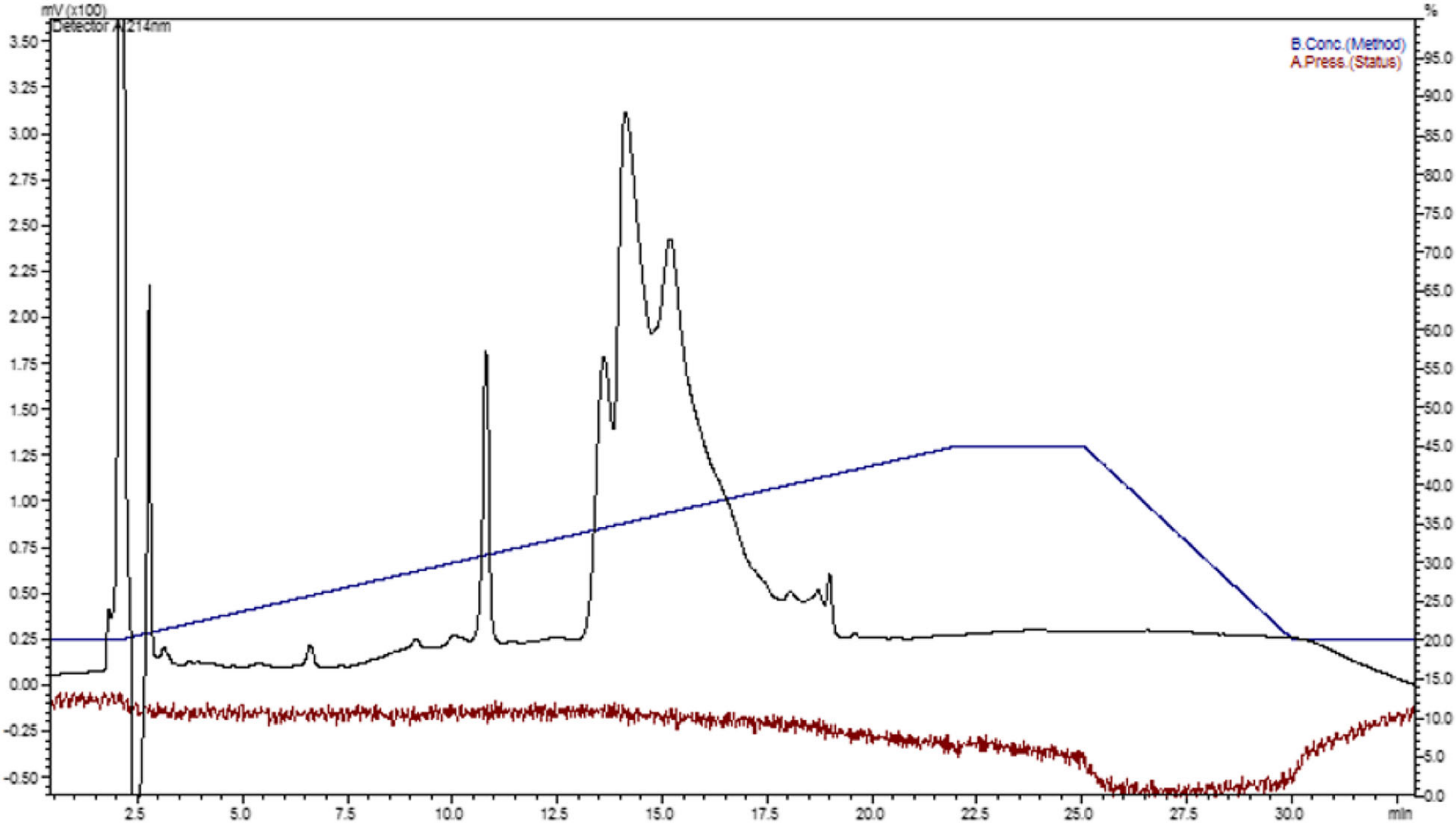
Reverse phase chromatogram of peak nine. Elution was performed with a gradient of B solution (90% acetonitrile/0.1% TFA/water) ranging from 20 to 50%, in 20 min, at a flow rate of 1 mL/min

This fraction was then further decomplexed by reverse phase chromatography on a C18 column, resulting in three peaks (Fig. 2).

### Mass spectrometry and *de novo* sequencing

**Fig. 3:**
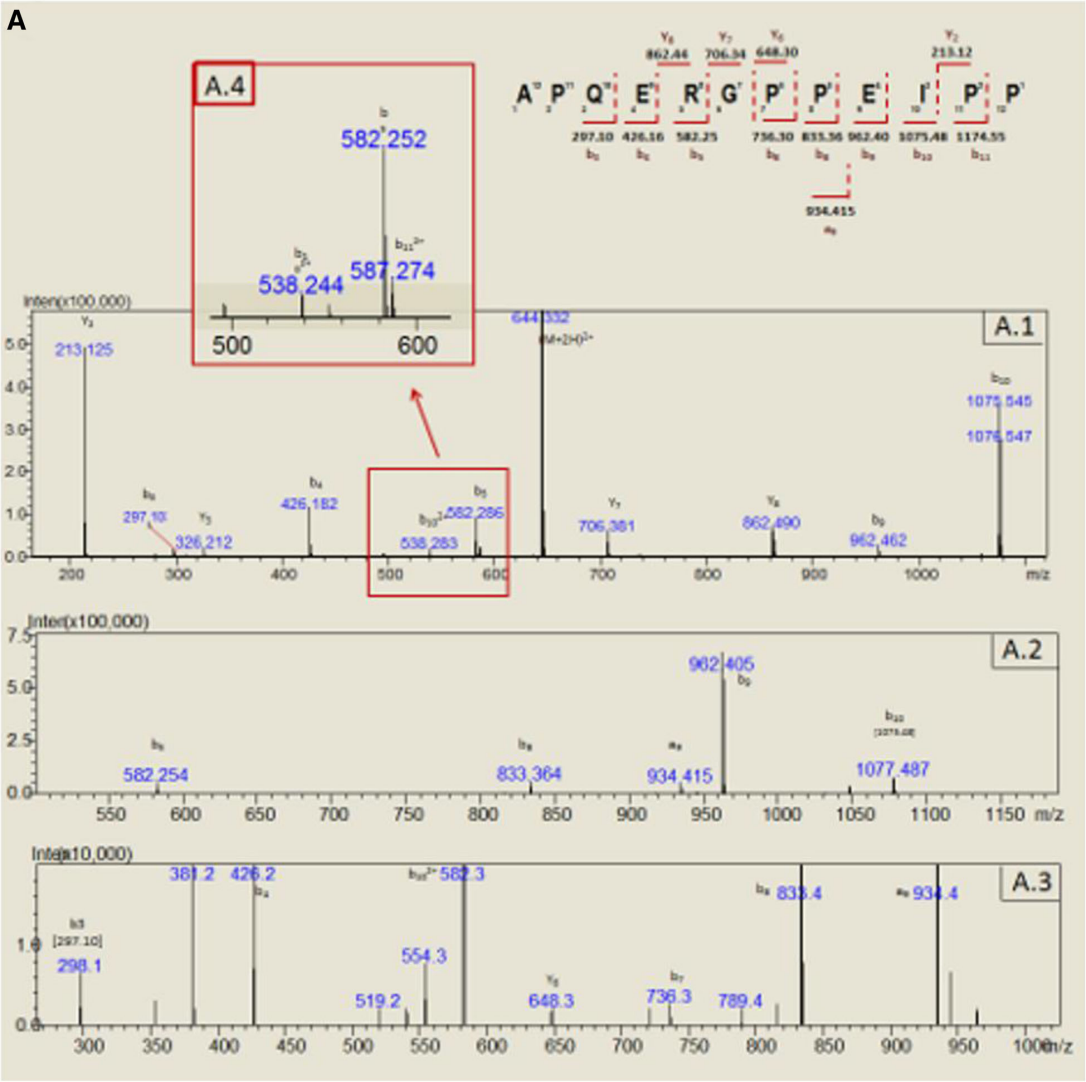
Representative CIF spectra of m/z = 644.30 [M + 2H]^2+^, (A.1) MS^2^ for m/z = 644.30, (A.2) MS^3^ for m/z = 1075.545, (A.3) MS^4^ for m/z = 962.405, (A.4) magnification of A.2 and respective annotations and sequence deduction

**Fig. 4:**
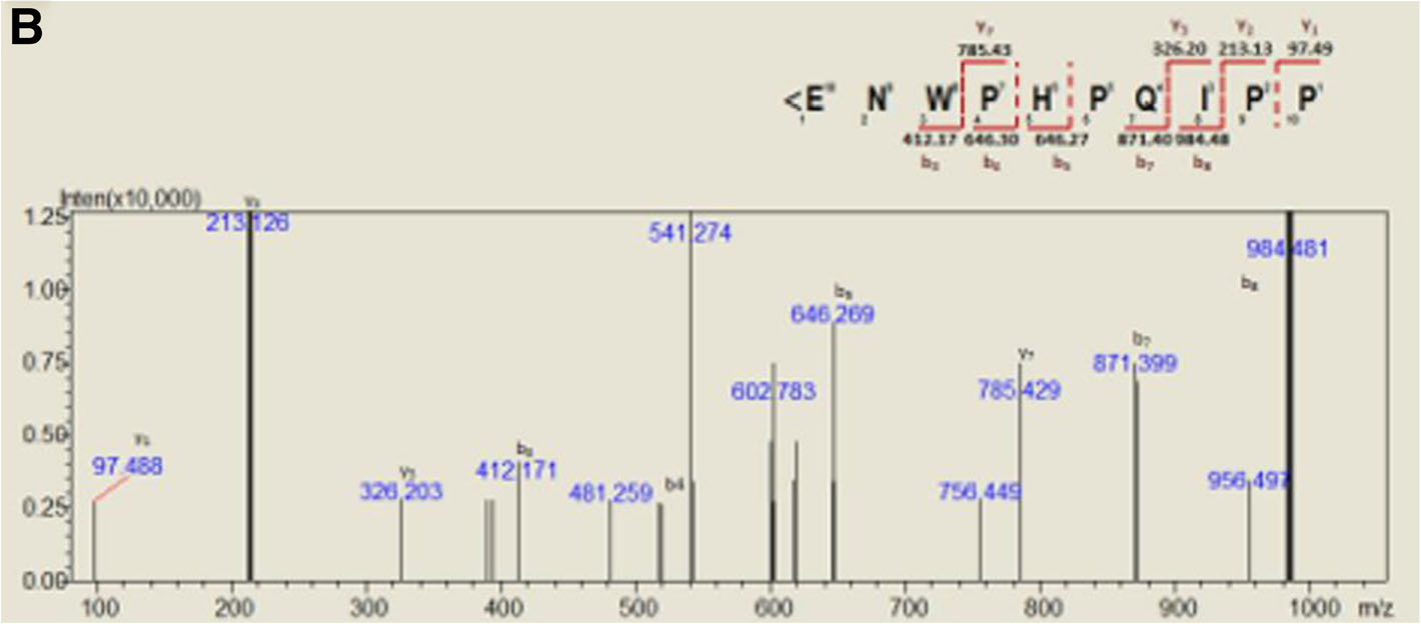
Representative CIF spectra of m/z = 984.48 [M + 2H]^2+^ and respective annotations and sequence deduction

The BPP-containing peak was analyzed by electrospray (MS; MS^2^ and MS^3^ were required for proper *de novo* sequencing). The interpreted annotated mass spectra are depicted below (Figs. 3 and 4).

The fragmentation of BPPs by collision-induced dissociation during electrospray tandem mass spectrometry analysis (ESI-MS/MS) generates a predominant signal at *m*/*z* 213.1 corresponding to the *y*-ion of the terminal Pro–Pro fragment [25]. This signature was observed in all spectra. The raw data were processed by Mascot (Matrix Science Inc., USA) and Peaks (Bioinformatics Solutions Inc., Canada). The *de novo* sequencing list of peptide was manually checked for accuracy.

### Inhibition assay

The hydrolysis rate of the synthetic substrate in the presence of different inhibitor concentrations resulted in a calculated Ki of 1 mM (data not shown).

### Bradykinin-potentiating activity in vivo

**Fig. 5:**
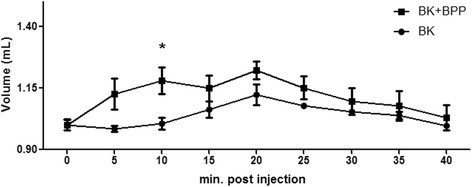
Rat paw edema induced by BK and BK in the presence of BPP-10 g-AP

Based on the *de novo* sequence of the native BPP, a synthetic peptide was purchased for activity assays. BK potentiating activity was investigated indirectly through the rat paw edema assay. Figure 5 shows the time course of rat paw edema after intraplantar injection of 40 ng/mL BK. The induced edema was detectable after 5 min and then declined at a constant rate over the next 40 min.

## Discussion

Many venom peptides mimic both functionally and structurally human molecules with physiological activity. These venom peptides target receptors and molecules, interfering in vital physiological processes such as hemostasis, coagulation and blood pressure. Their high specificity, low molecular mass (and therefore low immunogenicity), structural stability and relative ease of synthesis turn these peptides into a promising source of new drugs [26–28].

Envenomation by *Bitis* sp*.* often results in severe local damage, hypotension, coagulopathy, thrombocytopenia and spontaneous local bleeding and, in the absence of antivenom therapy, the accident can be fatal.

Proteomic analyses showed that metallopeptidases, serine peptidases, disintegrins, L-aminoacid oxidase, Kunitz inhibitors, phospholipases A_2_, cystatins and C-type lectins are present in *Bitis* venoms such as *B. arietans* and *B. g. rhinoceros* [27, 29]. Interestingly, the proteomic analysis of the venom of *B. gabonica* and *B. g. rhinoceros* demonstrated the presence of BPPs [30].

In this study, the low molecular mass fraction of *B. g. rhinoceros* venom was characterized by SEC, RP-HPLC, LC-MS/MS and bioassay. This strategy led to the identification of a novel non-canonical BPP, named BPP-10 g-AP.

The first ever described BPP, isolated from *Bothrops jararaca* venom, became the precursor for the development of anti-hypertensive drugs, such as Captopril® and Lisinopril® [22]. After the discovery of these first BPPs, similar proline-rich peptides were isolated from different snake venoms [31–34] and even frogs skin secretion [35, 36].

According to the kinetic assay, BPP-10 g-AP had inhibitory activity on ACE with a Ki of 1 μM, a much higher value than Captopril®, which displays a Ki of 0.046 μM [37]. Thus, although the inhibitory effect is believed to be associated with the presence of hydrophobic residues at the C-terminal region of the peptide, the N-terminal region (which usually starts with a pyroglutamic acid) may be important for affinity/specificity.

BK, as well as other kinins, presents several pharmacological actions such as vascular permeability alterations. Blood pressure drop induced by BK results from a decrease of vascular resistance in different organs such as heart, kidneys, intestine, skeletal muscles and liver [38].

Kinins trigger the increase of capillary flow, enabling the liquid exit from blood to tissues. This efflux can be facilitated by several factors such as increased vascular permeability and increase of the venous pressure, leading to liquid and proteins accumulation in the extravascular space, resulting in edema. Thus, one manner to investigate the activity of BK is to measure its edematogenic properties [37].

The synthetic BPP clearly induced an increase of the edematogenic activity of BK in our animal model. A previous study, using a similar approach, showed that the peptide KPP (kinin potentiating peptide, generated by tryptic digestion of human plasma proteins) and *B. jararaca* BPP9a (QWAQWPRPQIPP) induced intense and fast paw edema, similarly to our results [7].

Besides the new BPP, we identified one other BPP by *de novo* sequencing in *B. g. rhinoceros* venom (*<*ENWPRPQIPP), identical to *B. jararaca* BPP-10b. Interestingly, another peptide (m/z = 591.76 [M + 2H] ^2+^) that was only partially sequenced (data not shown) presented a typical y2 ion (PP) and displays a valine instead of isoleucine in the conserved IPP C-terminus. Calvete et al. [27] and Komori et al. [39] identified a similar C-terminal tripeptide in BPPs isolated from the venoms of *Vipera ammodytes meridionalis* and *V. a. aspis* respectively. Thus, so far, this BPP variant seems to be restricted to the Viperinae subfamily. The diversity of BPPs in a single venom has been ascribed to gene duplication and accelerated evolution within the BPP precursor gene [40]. It is therefore possible that the VPP motif has evolved after the split between Crotalinae and Viperinae*.* The occurrence of this VPP motif instead of the highly conserved IPP in the venoms of three dissimilar species strongly suggests that its presence is not accidental, indicating that there is some evolutionary advantage for the presence of BPPs with this sequence in snake venoms.

Snake venoms are composed by a mixture of components such as peptidases, phospholipases A_2_, myotoxins, neurotoxins and vasoactive peptides, which act synergistically, promoting a collapse of homeostasis [41]. These components interact with key physiological processes, leading to coagulopathies (peptidases, some phospholipases A_2_), tissue degradation (peptidases, phospholipases A_2_, myotoxins) and inhibition of the neuromuscular transmission [41]. In this context, BPPs play a key role in envenomation, by inducing severe hypotension and contributing for prey immobilization.

Small peptides, such as BPPs, are interesting and promising molecules from the biotechnological perspective. Especially when nature presents variations on the theme (such as BPP-10 g-AP N-terminal -AP-) providing a breath of fresh air in the continuous search of new anti-hypertensive molecules that would be either more potent or more selective to the C-domain.

## Conclusions

To the best of authors’ knowledge, this is the first identification of a canonical BPP in the *Bitis* genus. Such discovery is greatly dependent on both proper sample preparation and contemporary analytical techniques. Accordingly, BPP-10 g-AP amino acid sequence could only be deduced by mass spectrometry using the information gathered by MS^3^ and MS^4^, due to presence of two glutamic acids in the sequence that, according to our interpretation, impaired the ionization of the daughter fragments, yielding a non-informative spectrum (Fig. 3). It was only when MS^4^ was used that the full peptide sequence could be deduced. This emphasizes the importance of ion trap mass analyzers as tools for the discovery and characterization of new molecules.

## Abbreviations

ACE: Angiotensin-I converting enzyme
Ang II: Angiotensin II
BK: Bradykinin
BPP: Bradykinin-potentiating peptide
FRET: Fluorescence resonance energy transfer
MS: Mass spectrometry
MS^2^: 1^st^ Generation product ion spectra
MS^3^: 2^nd^ Generation product ion spectra
MS^4^: 3^rd^ Generation product ion spectra
SEC: Size exclusion chromatography
TFA: Trifluoroacetic acid
